# Formation of 4-hydroxynonenal and further aldehydic mediators of inflammation during bromotrichlorornethane treatment of rat liver cells

**DOI:** 10.1155/S0962935193000031

**Published:** 1993

**Authors:** Olaf Sommerburg, Tilman Grune, Sabine Klee, Fritz R. Ungemach, Werner G. Siems

**Affiliations:** 1 Institute of Biochemistry Medical Faculty (Charité) Humboldt University Berlin Hessische Str. 3-4 Berlin D(O)-1040 Germany; 2 lnstitute of Veterinary Pharmacology and Toxicology Free University Berlin Koserstr. Berlin 33 D-1000 Germany; 3 Herzog-Julius-Hospital Kurhausstr 13–17 Bad Harzburg D-3388 Germany

## Abstract

Bromotrichloromethane (CBrCl_3_) treatment is a model for studies on molecular mechanisms of haloalkane toxicity with some advantages compared with CCl_4_ treatment. The formation of 4-hydroxynonenal and similar aldehydic products of lipid peroxidation, which play a role as mediators of inflammatory processes, was clearly demonstrated in rat hepatocytes treated with CBrCl_3_. It may be assumed that haloalkane toxicity is connected with the biological effects of those inflammation mediatory aldehydic compounds.


Mediators of Inflammation, 2, 27–31 (1993)

### Formation of 4-hydroxynonenal and further aldehydic mediators of inflammation during bromotrichloromethane treatment of rat liver cells

**Olaf Sommerburg,[1] Tilman Grune,[1] Sabine Klee,[2] Fritz R. Ungemach[2] and Werner G. Siems[3,CA]**

[1]Institute of Biochemistry, Medical Faculty (Charité), Humboldt University Berlin, D(O)-1040 Berlin, Hessische Str. 3-4, Germany and [2]Institute of Veterinary Pharmacology and Toxicology, Free University Berlin, D-1000 Berlin 33, Koserstr., Germany
[3]Herzog-Julius-Hospital, D-3388 Bad Harzburg, Kurhausstr, 13–17, Germany

[CA] Corresponding Author

**Bromotrichloromethane ($CBrCl_3$) treatment is a model for studies on molecular mechanisms of haloalkane toxicity with some advantages compared with $CCl_4$ treatment. The formation of 4-hydroxynonenal and similar aldehydic products of lipid peroxidation, which play a role as mediators of inflammatory processes, was clearly demonstrated in rat hepatocytes treated with $CBrCl_3$. It may be assumed that haloalkane toxicity is connected with the biological effects of those inflammation mediatory aldehydic compounds.**

**Key words:** Aldehydes, Bromotrichloromethane, Haloalkanes, Hepatocytes, 4-Hydroxynonenal, Lipid peroxidation

### Introduction

Although pathophysiological and biochemical changes due to haloalkane intoxication have been investigated intensively by many laboratories, increasing exposure to toxic pollutants in the workplace and in the environment requires the continuation of studies on the molecular basis of haloalkane toxicity. Haloalkanes are also of importance in clinical practice because of the use of halothane and other halogenated compounds as inhalation anaesthetics, and a number of clinical cases of $CCl_4$ poisoning. Additionally 1,2-dibromomethane has been used as a fruit and grain fumigant.

Haloalkanes induce serious inflammatory reactions. Several reports exist on biochemical changes in plasma during halothane treatment characterizing these inflammatory reactions,[1,2] and on hepatitis and cirrhosis after halothane or $CCl_4$ intoxication.[3] The cytotoxic mechanisms of haloalkanes have been studied mostly in cell suspensions, organs or whole animals treated with $CCl_4$. $CBrCl_3$ was also used several times for investigations on haloalkane toxicity. $CCl_4$ is metabolized in a cytochrome P-450 dependent reaction which leads to the formation of the $^{\bullet}CCl_3$ radical. The $^{\bullet}CCl_3$ radical rapidly interacts with membrane lipids.[4–7] The $^{\bullet}CCl_3$ induced lipid peroxidation following $CCl_4$ treatment is considerably lower than that induced by $CBrCl_3$ treatment.[8] It is known that the C–Cl cleavage needs more energy (68 kcal/mol) than the cleavage of the C–Br bond (49 kcal/mol).[9] Koch *et al.*[8] investigated comparatively the capacity of $CCl_4$ and $CBrCl_3$ to induce lipid peroxidation. *In vitro*, $CBrCl_3$ induced lipid peroxidation was about 100 times more effective than the $CCl_4$ induced lipid peroxidation;[8] and *in vivo* was three times more effective. That favours the $CBrCl_3$ model for studies on cytotoxic effects of haloalkanes in comparison with the $CCl_4$ model.

The most common parameter for the measurement of lipid peroxidation and its products is the generation of malondialdehyde (MDA) in exposed systems. However, the various procedures of MDA determination produce different results.[10] Furthermore, those methods are rather unspecific for the free metabolite MDA.[11] The determination of other aldehydes—e.g. 4-hydroxynonenal (HNE), as the main representative of 4-hydroxyalkenales—is a more specific and sensitive method for the estimation of lipid peroxidation. It is known that 4-hydroxynonenal and some other aldehydic products of lipid peroxidation are much more effective as inflammatory agents, e.g. as chemoattractive substances, than MDA.[12] Poli *et al.* determined the formation of various aldehydic products of lipid peroxidation during $CCl_4$

© 1993 Rapid Communications of Oxford Ltd treatment in hepatocyte suspensions.[13] MDA formation was measured in $CBrCl_3$ treated cells too,[4,5,14] but there are no data on the formation of 4-hydroxyalkenales and further aldehydic products of lipid peroxidation except MDA in $CBrCl_3$ treated cells, organs or animals. This paper deals with the determination of such highly reactive mediators of inflammation in the $CBrCl_3$ intoxication model.

### Materials and Methods

*Chemicals:* Aldehyde standards (dinitrophenylhydrazones) were prepared in the laboratory of Prof. H. Esterbauer, Karl-Franzens University Graz, Austria. The solvents *n*-hexane, acetonitrile, dichloromethane, methanol and benzene, and the TLC plates (silica gel 60, 0.2 mm thickness) were from Merck (Darmstadt, Germany). 2,4-Dinitrophenylhydrazine (DNPH) was obtained from Union Chimique (Brussels, Belgium). DNPH was dissolved in 5 ml of 1 M hydrochloric acid, extracted with 3 × 5 ml of *n*-hexane, then adjusted to 1.8 mM solution with 1 M hydrochloric acid by means of absorbance measurement at 378 nm. Collagenase (*Clostridium histolyticum*) was from Boehringer (Mannheim, Germany). All other reagents were purchased from Sigma Chemie GmbH (Deisenhofen, Germany). Thiobarbituric acid and malondialdehyde diethylacetal were obtained from Aldrich Chemie GmbH (Steinheim, Germany).

*Cell preparation:* Male Wistar H-strain rats with a body weight of about 230 g were used. Hepatocytes were prepared according to the method given in Berry and Friend[15] with modifications given in Vincent[16]. Starved rats (24 h) were anaesthetized by an intraperitoneal injection pentobarbital (30 mg/kg body wt.). As perfusion medium a Krebs–Henseleit bicarbonate buffer gassed with $O_2$:$CO_2$ (19:1) was used.

*Cell incubation:* The cell viability was determined by cell staining with trypan blue. Only hepatocyte suspensions with more than 85% of viable cells excluding trypan blue were used for experiments. The cell suspensions were adjusted to a cytocrit of 2% (v/v) ($5.2 \times 10^6$ cells/ml) with Krebs–Henseleit buffer as the incubation medium. The cells were incubated at 37°C under continuous gyratory shaking. $CBrCl_3$ dissolved in ethanol was added in a final concentration of 1 mM. The controls were treated with the same amount of ethanol (1% final concentration). The aldehydic formation in hepatocyte suspensions induced by 1 mM $CBrCl_3$ was compared with aldehyde formation in hepatocyte suspensions which were incubated in presence of 1 mM $CCl_4$ under the same conditions of preparation, incubation, extraction and analysis.[13]

*Determination of 4-hydroxynonenal and further aldehydes:* The detection of aldehydes was performed according to Esterbauer *et al.*[17] and Poli *et al.*[13]

*Derivatization of aldehydes with DNPH:* 2.5 ml of the cell suspension reacted with 2.5 ml of DNPH solution (1.8 mM in 1 N HCl) in the presence of butylated hydroxytoluene (BHT) (final concentration 10 mM) for 2 h in the dark. Thereafter, the sample was kept on ice for 1 h in the dark, then extracted three times with 8 ml of dichloromethane and centrifuged at 900 × ***g***. The supernatant was evaporated on a rotary evaporator to dryness at 33°C. The residue, dissolved in 1 ml of dichloromethane, was transferred into small vials for spotting on TLC plates.

*Thin-layer chromatography:* The TLC plates were developed with dichloromethane in comparison with known standards. By this method according to details given in Esterbauer *et al.*[17] the dinitrophenylhydrazones of carbonyl compounds were separated into three zones. Zone I contained the 4-hydroxyalkenales. Zone I and Zone III were scraped off, each extracted three times with 10 ml of methanol and evaporated to dryness. The residue was dissolved in 1 ml of methanol.

*HPLC equipment and chromatographic conditions:* A HPLC system from Perkin Elmer was used consisting of a M410 pump system, a LC 95 variable wavelength detector, a LCI-100 integrator and a Rheodyne injector. For some determinations a Photodiode-Array-Detector (Hewlett–Packard) was used additionally.[18] The eluent for the isocratic separation was methanol–water (4:1, v/v), at a flow rate of 1 ml/min. A Nucleosil 5C18 column (Macherey, Nagel & Co., Düren, Germany, 250 × 4.0 mm I.D.) was used, with a 20 × 4.0 mm I.D. precolumn. Peak identification was performed by comparison of the retention times of peaks of biological extracts and of standard extracts, and also by the coelution of biological extracts with the reference compound and by comparison of the spectra of the analytes. Quantification of HNE was achieved by separating HNE–DNPH standard solutions of different concentrations. Quantification of further aldehydes was also performed using different concentrations of their dinitrophenylhydrazone derivatives.

*Recovery:* The recovery was determined using a HNE standard solution (10 $\mu$M) and amounted to $32 \pm 5\%$ (mean ± S.D.). The value of HNE recovery was highly reproducible. Recoveries of other carbonyls were quantified in an analogous manner. All recoveries were between 25 and 40%. The values of HNE and other aldehydes were corrected to 100%.

*Determination of malondialdehyde (MDA):* MDA was measured according to Wong *et al.*[19] A 200 $\mu$l sample of cell suspension was mixed with 0.75 ml phosphoric acid (0.44 mol/l), 0.25 ml thiobarbituric acid (42 mmol/l) and 0.3 ml water and boiled for 60 min. The reaction was stopped by cooling the samples in an ice bath. Immediately before HPLC analysis an equal volume of 1 M NaOH was added followed by centrifugation. The HPLC system consisted of a Waters 510 pump, a Shimadzu fluorescence detector RF-530 (525/550 nm) and a C-R6A integrator. The column was a Supelcosil 150 × 4 mm LC-18-S (5 $\mu$M). As eluent, a 50 mM potassium phosphate buffer solution (pH 6.8) with 40% methanol was used.

### Results

In this study of HNE, MDA and further aldehydic products of lipid peroxidation could be detected and quantitatively evaluated in the samples of hepatocyte suspensions. There were no differences between HNE levels of controls and $CBrCl_3$ treated cells at time zero of the experiments (Fig. 1). The HNE concentration of control suspension was constant during 1 hr of incubation. The HNE level was about 200 nmol/l suspension (0.2 $\mu$M). In the case of $CBrCl_3$ treatment, 5 min after the addition of haloalkane to the cell suspension the HNE level was markedly increased. The HNE value at this time point was about 600 nmol/l cell suspension. This three-fold increase in the HNE level was maintained throughout the whole experiment. There was even a trend for a further slight increase between 45 and 60 min after $CBrCl_3$ addition.

Figure 2 shows the changes of MDA concentration in $CBrCl_3$ treated hepatocyte suspensions. The level of MDA at time zero was about 800 nmol/l cell suspension (0.8 $\mu$M). There was a slight decrease of MDA level in control suspensions during the first 15 min of incubation. Therefore, the control values of MDA level were in the range of 0.5 to 0.8 $\mu$M. $CBrCl_3$ treatment increased the MDA concentration within 15 min (about twice the initial value). Table 1 represents data on the further aldehydic products of lipid peroxidation during 60 min of $CBrCl_3$ treatment of hepatocyte suspensions. It was possible to detect low amounts of such aldehydic compounds in $CBrCl_3$ treated cells. In Fig. 3 the accumulation of MDA, HNE, pentanal and heptanal in $CCl_4$ and in $CBrCl_3$ treated hepatocyte suspensions is compared.

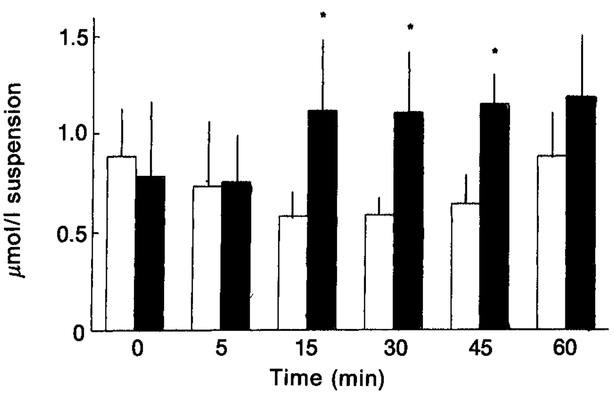

FIG. 2 Concentration of thiobarbituric acid—reactive substances in hepatocyte suspensions after bromotrichloromethane treatment. Symbols are: □ Control (addition of ethanol), ■ bromotrichloromethane treatment (1 mM). Values are given as mean ±S.D. (*$p < 0.05$ as compared with controls).

**Table 1.** Levels of aldehydic products of lipid peroxidation in hepatocyte suspensions treated 60 min with 1 mM $CBrCl_3$. Values are given as nmol/l cell suspension; mean ± S.D. (six experiments)

| Aldehyde | 0 min | 60 min |
|---|---|---|
| MDA | 782 ± 385 | 1180 ± 355 |
| HNE | 190 ± 70 | 900 ± 160 |
| Hexadienal | 2.4 ± 1.0 | 6.3 ± 1.8 |
| Heptadienal | 0.6 ± 0.6 | 6.0 ± 1.0 |
| Nonadienal | 7.5 ± 2.6 | 11.7 ± 2.2 |
| Decadienal | 13.2 ± 4.0 | 24.2 ± 4.4 |
| Pentanal | 9.3 ± 1.7 | 27.1 ± 2.9 |
| Heptanal | 26.0 ± 5.4 | 40.7 ± 4.0 |

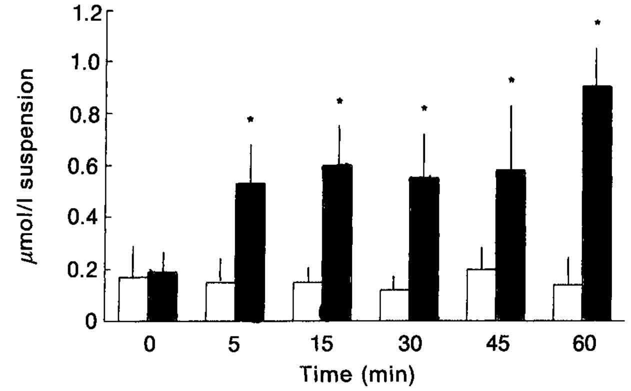

FIG. 1 Concentration of 4-hydroxynonenal in hepatocyte suspensions after bromotrichloromethane treatment. Symbols are: □ Control (addition of ethanol), ■ bromotrichloromethane treatment (1 mM). Values are given as mean ± S.D. (*$p < 0.05$ as compared with controls).

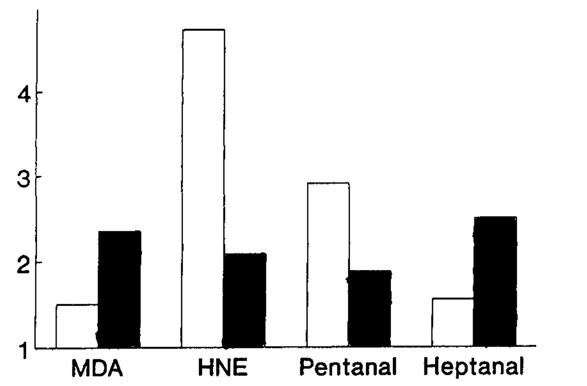

FIG. 3 Concentration ratios of aldehydes after 60 min of incubation with □ $CBrCl_3$ and ■ $CCl_4$ (see Reference 13) compared with the initial concentration in the same suspension.

### Discussion

*Pathophysiological role of aldehydic products of lipid peroxidation:* There is increasing evidence that aldehydes generated during the process of lipid peroxidation are causally involved in some of the pathophysiological effects associated with oxidative stress in cells and tissues.[20] Unlike reactive free radicals, aldehydes are rather long-lived and can therefore diffuse from the site of their origin, i.e. membranes, and reach and attack targets intracellularly or extracellularly which are distant from the initial free radical event. HNE and similar aldehydic products of lipid peroxidation exhibit a wide spectrum of cytotoxic effects as well as mutagenic and genotoxic effects.[20–23]

Since HNE, and other aldehydes, are formed during the acute phase of inflammation they may play a role as mediators in the inflammatory process.[20] HNE effects produced even at HNE concentrations of 0.1 $\mu$M or less include stimulation of chemotactic oriented migration of neutrophils,[12,24,25] modulation of adenylate cyclase activity, weak stimulation of guanylate cyclase[26] and stimulation of phospholipase C.[27] Phospholipase C is important for signal transduction by G-proteins. Stimulation of phospholipase C leads to increased hydrolysis of phosphatidyl-4,5-bisphosphate with concomitant formation of diacylglycerol and inositol triphosphate. Diacylglycerol acts synergistically with inositol triphosphate in the calcium dependent activation of protein kinase C. Inositol triphosphate opens calcium channels, whereas diacylglycerol increases the calcium affinity of protein kinase C. Protein kinase C plays an important role in the control of cell division, cell proliferation and other cellular functions. Phospholipase C stimulation may also be responsible for the chemotactic effects of 4-hydroxynonenal since it is known that the chemotactic signal of f-met peptides also results in an activation of phospholipase.[28]

4-Hydroxyalkenals induce the oriented migration and morphological polarization of neutrophils in a concentration range of $10^{-6}$ to $10^{-12}$ M depending on the chain length of the aldehyde. The most effective aldehyde was not HNE but 4-hydroxyoctenal with a maximum chemotactic activity at $10^{-11}$ M. For HNE the effective doses for chemotactic effects and for activation of phospholipase C are between $10^{-6}$ and $10^{-9}$ M.[25]

Cytotoxic haloalkane effects depend on the formation and action of free radicals initiating lipid peroxidation.[4,5,29] The formation of HNE and similar aldehydic products of lipid peroxidation in hepatocytes treated with $CCl_4$ was published by Poli *et al.*[13] In the present study the formation of such compounds could also be demonstrated for $CBrCl_3$.

*Critical aspects of methodological approach:* For the determination of long chain aldehydes a number of methods, such as the dinitrophenylhydrazine method, the direct determination by HPLC, the cyclohexadione method and the determination by GC–MS, are described in Esterbauer and Zollner[30]. The determination of a wide spectrum of aliphatic aldehydes is only possible, using the dinitrophenylhydrazine and the cyclohexadione method, with comparable results in recovery and reproducibility. The literature results for reproducibility and low recovery (about 40%) are in accordance with those achieved in this study.[30,31] The determination of the relatively low recoveries for each aldehyde allows the determination of the real biological steady state concentration in the cell suspension. Nevertheless, the low recovery and therefore a certain inaccuracy of methods which were available for aldehyde analysis modify the quantitative aspect of those measurements.

*Comparison of formation of aldehydes as mediators of inflammation in $CCl_4$ and $CBrCl_3$ toxicity:* The initial levels of MDA and of HNE at zero time were in the same range as concentrations reported by other authors in hepatocyte suspensions.[13] In contrast to $CCl_4$ treated cells the aldehyde levels increased very rapidly, i.e. within the first minutes following $CBrCl_3$ addition to the hepatocytes. However, the increases of MDA and HNE levels in hepatocyte suspensions treated with $CBrCl_3$ were in the same range as in those hepatocyte suspensions treated with equimolar concentrations of $CCl_4$.[13] The increase of some aldehydic products of lipid peroxidation within 1 h of treatment with $CCl_4$ or $CBrCl_3$ in comparison to initial levels is demonstrated in Fig. 3. The most interesting point of this comparison seems to be the excessive increase of HNE as a very cytotoxic and chemotactic compound at $CBrCl_3$ loading compared with $CCl_4$ loading.

The generation of aldehydic products of lipid peroxidation other than MDA and HNE could be demonstrated for $CBrCl_3$ incubations (Table 1) but at markedly lower concentrations than those for MDA and HNE. Nevertheless, the amounts of these aldehydes and of HNE which were produced even during short-term experiments are high enough for the initiation of chemotactic activity towards neutrophils.

It should be mentioned that the determined aldehyde concentrations represent, of course, the steady state concentrations, resulting from a continuous formation of the aldehydes and a consumption of these compounds by the hepatocytes. The HNE metabolism—including that in hepatocytes—was investigated in several studies.[13,32–35] In general the maximal capacity of tissues to metabolize aldehydes, especially HNE, is in the order of several micromoles per gram wet weight per minute, which is about six orders higher than the accumulation within the first minutes after haloalkane treatment.

The specific pattern of aldehydes formed by $CBrCl_3$ and $CCl_4$ is different, whereas the overall increase in MDA and HNE is comparable for both compounds. The differences in lifetime, reactivity and 'consumption' rate of aldehydes may implicate differences in the pattern of cell and tissue damage if a different aldehyde pattern occurs. There is the possibility that in $CBrCl_3$ induced liver damage 4-hydroxynonenal and similar aldehydic products of lipid peroxidation are involved in cell damage and act as mediators of inflammatory processes. The relation between formation and 'consumption' of aldehydes should be investigated in further experiments.

### References

1. Tsuchiya M, Okimasu E, Ueda W, Hirakawa M, Utsumo K. Halothane, an inhalation anesthetic, activates protein kinase C and superoxide generation by neutrophiles. *FEBS Lett* 1988; **242**: 101–105.
2. Fukai F, Nishizawa S, Kurano M, Taniguchi K, Katayama T. Carbon tetrachloride-induced alteration of glutathione S-transferase in rat liver cytosol and plasma. *J Clin Biochem Nutr* 1989; **6**: 175–185.
3. Arii S, Monden K, Itai S *et al.* Depressed function of Kupffer cells in rats with $CCl_4$-induced liver cirrhosis. *Res Exp Med* 1990; **190**: 173–182.
4. McGirr LG, Khan S, Lauriault V, O'Brien PJ. Molecular mechanisms for bromotrichloromethane cytotoxicity in isolated rat hepatocytes. *Xenobiotica*; 1990; **20**: 933–943.
5. Ungemach FR. Pathobiochemical mechanisms of hepatocellular damage following lipid peroxidation. *Chem Phys Lipids* 1987; **45**: 171–205.
6. Albano E, Carini R, Parola M, Bellomo G, Poli G, Dianzani MU. Increase in cytosolic free calcium and its role in the pathogenesis of hepatocyte injury induced by carbon tetrachloride. In: Poli G, Cheeseman KH, Dianzani MU, Slater TF, eds. *Free Radicals in the pathogenesis of liver injury.* Oxford, New York: Pergamon Press, 1989; 45–55.
7. Danni O, Aragon M, Biasi F, Chiarpotto E, Comoglio A, Poli G. Hepatocyte damage induced by carbon tetrachloride and 1,2-dibromoethane mixture: role of lipid peroxidation and GSH depletion. In: Poli G, Cheeseman KH, Dianzani MU, Slater TF, eds. *Free Radicals in the pathogenesis of liver injury.* Oxford, New York: Pergamon Press, 1989; 63–73.
8. Koch RR, Glende EA, Rechnagel RO. Hepatotoxicity of bromotrichloromethane—bond dissociation energy and lipoperoxidation. *Biochem Pharmacol* 1974; **23**: 2907–2915.
9. Slater TF. *Free radical mechanisms in tissue injury.* London: Pion, 1972.
10. Grune T, Siems W, Esterbauer H. Comparison of different assays for malondialdehyde using thiobarbituric acid. *Fresenius J Anal Chem* 1992; **343**: 135.
11. Janero DR. Malondialdehyde and thiobarbituric acid-reactivity as diagnostic indices of lipid peroxidation and peroxidative tissue injury. *Free Rad Biol Med* 1990; **9**: 515–540.
12. Curzio M. Interaction between neutrophils and 4-hydroxyalkenales and consequences on neutrophil motility.*Free Rad Res Comms* 1988; **5**: 55–66.
13. Poli G, Dianzani MU, Cheeseman KH, Slater TF, Lang J, Esterbauer H. Separation and characterization of the aldehydic products of lipid peroxidation stimulated by carbon tetrachloride or ADP-iron in isolated rat hepatocytes and rat liver microsomal suspensions. *Biochem J* 1985; **227**: 629–638.
14. Köster-Albrecht D, Kappus H, Remmer H. Lipid peroxidation and cell damage in isolated hepatocytes due to bromotrichloromethane. *Toxicol Appl Pharmacol* 1978; **46**: 499–505.
15. Berry MN, Friend DS. High-yield preparation of isolated rat liver parenchymal cells. *J Cell Biol* 1969; **43**: 506–520.
16. Vincent M-F. The pathway of adenine nucleotide catabolism and its control in isolated rat hepatocytes subjected to anoxia. *Biochem J* 1982; **202**: 117–123.
17. Esterbauer H, Cheeseman KH, Dianzani MU, Poli G, Slater TF. Separation and characterization of the aldehydic products of lipid peroxidation stimulated by ADP-$Fe^{2+}$ in rat liver microsomes. *Biochem J* 1982; **208**: 129–140.
18. Werner A, Siems W, Grune T, Schreiter C. Interrelation between nucleotide degradation and aldehyde formation in red blood cells. *J Chromatogr* 1990; **507**: 311–319.
19. Wong SHJ, Knight JA, Hopfer SM, Zaharia O, Leach CN, Sunderman FW. Lipoperoxides in plasma as measured by liquid-chromatographic separation of malondialdehyde–thiobarbituric acid adduct. *Clin Chem* 1987; **33**: 214–220.
20. Esterbauer H, Schaur RJ, Zollner H. Chemistry and biochemistry of 4-hydroxynonenal, malondialdehyde and related aldehydes. *Free Rad Biol Med* 1991; **11**: 81–128.
21. Esterbauer H, Zollner H, Schaur RJ. Hydroxyalkenales; Cytotoxic products of lipid peroxidation. *ISI Atlas Sci Biochem* 1988; **1**: 31–317.
22. Schaur RJ, Zollner H, Esterbauer H. Biological effects of aldehydes with particular attention to 4-hydroxynonenal and malondialdehyde. In: Vigo-Pelfrey C, ed. *Membrane lipid peroxidation* vol. III. Boca Raton, Ann Arbor, Boston: CRC Press, 1991; 141–163.
23. Eckl P, Esterbauer H. Genotoxic effects of 4-hydroxyalkenals. *Adv Biosci* 1990; **76**: 141–157.
24. Curzio M, Esterbauer H, Dianzani MU. Chemotactic activity of hydroxyalkenals on rat neutrophils. *Int J Tissue React* 1985; **7**: 137–142.
25. Curzio M, Esterbauer H, DiMauro C, Cecchini G, Dianzani MU. Chemotactic activity of the lipid peroxidation product 4-hydroxynonenal and homologous hydroxyalkenals and consequences on neutrophil motility. *Free Rad Res Communs* 1988; **5**: 55–66.
26. Dianzani MU, Paradisi L, Barrera G, Rossi MA, Parola M. The action of 4-hydroxynonenal on the plasma membrane enzymes from rat hepatocytes. In: Baumont PC, Deeble DJ, Parsons BJ, Rice-Evance C, eds. *Free Radicals, metal ions and biopolymers.* London: Richelieu-Press, 1989; 329–346.
27. Rossi MA, Fidale F, Garramone A, Esterbauer H, Dianzani MU. Effect of 4-hydroxyalkenals on hepatic phosphatidylinositol-4,5-biphosphate-phospholipase C. *Biochem Pharmacol* 1990; **39**: 1715–1719.
28. Stryer L, Bourne HR. G-proteins—a family of signal transducers. *Ann Cell Biol* 1986; **2**: 393–419.
29. Recknagel RO, Goshal AK. Lipid peroxidation as a vector in carbon tetrachloride hepatotoxicity. *Lab Invest* 1982; **15**: 132–138.
30. Esterbauer H, Zollner H. Methods for determination of aldehydic lipid peroxidation products. *Free Rad Biol Med* 1989; **7**: 197–203.
31. Esterbauer H, Cheeseman KH. Determination of aldehydic lipid peroxidation products: Malonaldehyde and 4-hydroxynonenal. In: *Methods in Enzymology* Vol. 186, Academic Press, 1990; 407–421.
32. Siems WG, Zollner H, Esterbauer H. Metabolic pathways of the lipid peroxidation product 4-hydroxynonenal in hepatocytes—quantitative assessment of an antioxidative defense system. *Free Rad Biol Med* 1990; **9**: 110.
33. Siems WG, Zollner H, Grune T, Esterbauer H. Qualitative and quantitative determination of metabolites of the lipid peroxidation product 4-hydroxynonenal from hepatocytes, enterocytes and tumor cells. *Fresenius J Anal Chem* 1992; **343**: 75–76.
34. Grune T, Siems W, Kowalewski J, Zollner H, Esterbauer H. Identification of metabolic pathways of the lipid peroxidation product 4-hydroxynonenal by enterocytes of rat small intestine. *Biochem Int* 1991; **25**: 963–971.
35. Siems WG, Grune T, Beierl B, Zollner H, Esterbauer H. The metabolism of 4-hydroxynonenal, a lipid peroxidation product, is dependent on tumor age in Ehrlich ascites cells. In: Emerit I, Chance B, eds. *Free radicals and aging.* Basel, Birkhäuser Verlag, 1992; 124–135.

ACKNOWLEDGEMENTS. This study was financially supported by the Deutsche Forschungsgemeinschaft (DFG), Bad Godesberg, Bonn, Germany and by a grant of the Medical Faculty (Charité) Research Commission, Berlin, Germany.

**Received 17 August 1992; accepted in revised form 21 October 1992**
